# Permanent left bundle branch area pacing utilizing intracardiac echocardiogram

**DOI:** 10.1186/s12872-020-01649-0

**Published:** 2020-08-18

**Authors:** Xiang-Fei Feng, Peng-Pai Zhang, Bo Liu, Yan Zhao, Qiu-Fen Lu, Yi-Gang Li

**Affiliations:** Department of Cardiology, Xinhua Hospital, School of Medicine, Shanghai Jiao Tong University, 1665#, KongJiang Road, Shanghai, 200092 China

**Keywords:** Left bundle branch, Pacing, Intracardiac echocardiogram, Localization, Nine-grid system

## Abstract

**Background:**

Recently, left bundle branch area pacing (LBBAP) has been shown to be feasible. However, the right ventricular (RV) implantation site for LBBAP remains elusive. We believe that the RV implantation site should be located at the posteromedial basal septum, and in this paper, we propose a new method to help guide lead implantation. The aim of this study is to demonstrate the feasibility of the proposed method.

**Methods:**

The RV implantation site was positioned by a combination of a nine-grid system on fluoroscopy and the use of intracardiac echocardiogram (ICE) and then verified by ICE.

**Results:**

Fifteen patients were enrolled for LBBAP using our method. The acute success rate was 86.7% (13/15), which demonstrated that our method is useful for assisting with lead implantation. According to ICE, the distance between the implantation site and apex (the front) and the distance between the implantation site and tricuspid annulus (the back) were 44.9 ± 10.7 and 33.2 ± 10.4 mm, respectively, and the ratio of the front and the back was 1.57 ± 0.80. The distance between the implantation site and the front junction point of the left-right ventricle (the upper) and the distance between the implantation site and the back junction point (the lower) were 33.4 ± 10.6 and 24.5 ± 10.2 mm, respectively. The ratio of the upper to the lower was 1.76 ± 1.36. These results suggest that the implantation site was at the posteromedial basal septum. The width of the QRS duration increased from 110.4 ± 33.1 ms at baseline to 114.1 ± 16.1 ms post LBBAP (*P* > 0.05). The operation time was 133 ± 32.9 min. The time of X-ray fluoroscopy was 21.2 ± 5.9 min. The mean time for lead positioning during LBBAP was 33.8 ± 16.6 min. During a follow-up of 3 months, the LBB capture threshold remained stable in 12 patients, except for one patient who had an increase in the LBB capture threshold to 3.0 v/0.4 ms.

**Conclusions:**

Our preliminary results indicate that the posteromedial basal septum could be seen as the implantation site for LBBAP. As a technique for LBBAP, ICE is a useful method for assisting with lead implantation. It is feasible and safe to use a nine-grid system combined with ICE for LBBAP.

## Background

His-Purkinje system pacing has been proposed as the most physiologic mode of ventricular pacing. Previous studies have demonstrated that bundle branch block could be corrected by pacing at the distal His bundle and validated the safety and clinical benefits in patients with various cardiac diseases [1–4].

However, previous studies have shown that the His bundle pacing threshold significantly increased with time, even went over the capture threshold, to correct the left bundle branch (LBB) block [3, 5]. Other challenging issues remain, including the long implantation time, the long fluoroscopic time, and the high and unstable pacing threshold, especially in patients who have pathological disease in the conduction system [2–4, 6].

It is known that after penetrating the membranous atrioventricular (AV) septum, conductive fibres of the LBB are spread beneath the endocardium of the interventricular septum (IVS) over a relatively large area, which offers an easier approach for pacing the LBB [7]. Recently, left bundle branch area pacing (LBBAP) has been shown to be feasible by advancing the lead transvenously, deep into the IVS to pace both the LBB and adjacent ventricular tissues [8, 9]. LBBAP does not require high pacing output to achieve the correction of LBB block, as in the case of His bundle pacing. Moreover, LBB pacing may avoid later adverse impacts on the proximal His bundle or AV node caused by the progression of AV conduction delay, and LBB pacing also provides more anatomical space for AV node ablation [10].

However, the right ventricular (RV) implantation site for LBBAP remains elusive. We believe that the implantation site at the RV side of the IVS and the implantation direction of the lead tip in the septum are two essential elements to warrant successful LBBAP. The RV implantation site is usually identified on the basis of the His bundle potential signal, so the dual-lead method is helpful in finding the implantation site [11], but it is time consuming and difficult for a beginner to search for this signal. His bundle may sometimes be damaged, or even blocked during the searching process by new implanters.

LBB originates in the branching portion underneath the membranous septum [12]; as a result, we believe the RV implantation site of LBBAP should be located around the posteromedial basal septum. In this study, we propose a new method (a combination of nine-grid system on fluoroscopy and use of ICE for lead implantation) to help guide lead implantation to the desired site. Real-time ICE has become available [13] and is a useful tool for identifying pacing sites and monitoring lead orientation. The aim of this report is to demonstrate the feasibility of the proposed method.

## Methods

This study was approved by the Ethics Committee of Xinhua Hospital Affiliated with Shanghai Jiaotong University School of Medicine (approval number: XHEC -D-2019-055) and performed in accordance with the Declaration of Helsinki. The RV implantation site was positioned by our proposed methods and then verified by ICE.

### Patient selection

Patients who had indications for pacing therapy according to the 2013 ESC/EHRA Guidelines and underwent ICE guided LBBAP in our centre from Nov 1, 2018 to Jun 31, 2019 were enrolled, and the clinical data were analysed retrospectively. All patients submitted written informed consent and demonstrated an understanding of LBBAP as a nonstandard approach to achieve physiologic pacing.

### Lead implantation technique

As previously described [14–16], a Select Site C315 His sheath and a Select Secure 3830 pacing lead (Medtronic Inc., Minneapolis, MN, USA) were placed into the RV outlet tract (OT) via the left axillary (or subclavian) vein. The right ventricular septal location for LBBAP was identified using both anatomical localization and pacing localization. Once this site was identified, the pacing lead was advanced deep into the septum while controlling the lead orientation and depth using two-way monitoring and monitoring unipolar pacing impedance, electrogram characteristics and paced QRS morphology. Once the LBBAP success criteria were met, the implantation site, the angle and depth of the lead into the IVS are assessed with the help of ICE and 1–2 ml of contrast injected through the delivery sheath.

### ICE advancing technique

An ICE catheter (SOUNDSTAR Catheter, Biosense-Webster) was advanced through the right femoral vein towards the mid-right atrium (RA). The basic rule to advance the ICE catheter in vascular or cardiac chambers is to maintain an echographic clear space (black) ahead of the catheter and avoid pushing when an echogenic space (white) is ahead of the catheter [17].

The ICE catheter is initially in “home” view in the mid-RA, and the transducer in a neutral position is facing the tricuspid valve (TV). From the home view, clockwise rotation to the six o’ clock of the catheter brings into view the upper and lower right pulmonary veins; then, the catheter is “P” flexed and could be advanced gently into the RV. Once the catheter tip passes through the TV, the deflection is released gently until the tip points to the RV apex, and a view of the inferior RV is obtained. From this RV view, the catheter is gradually rotated clockwise and “L” flexed to image the long axis view of the left ventricle (LV) and IVS [17].

**Fig. 1:**
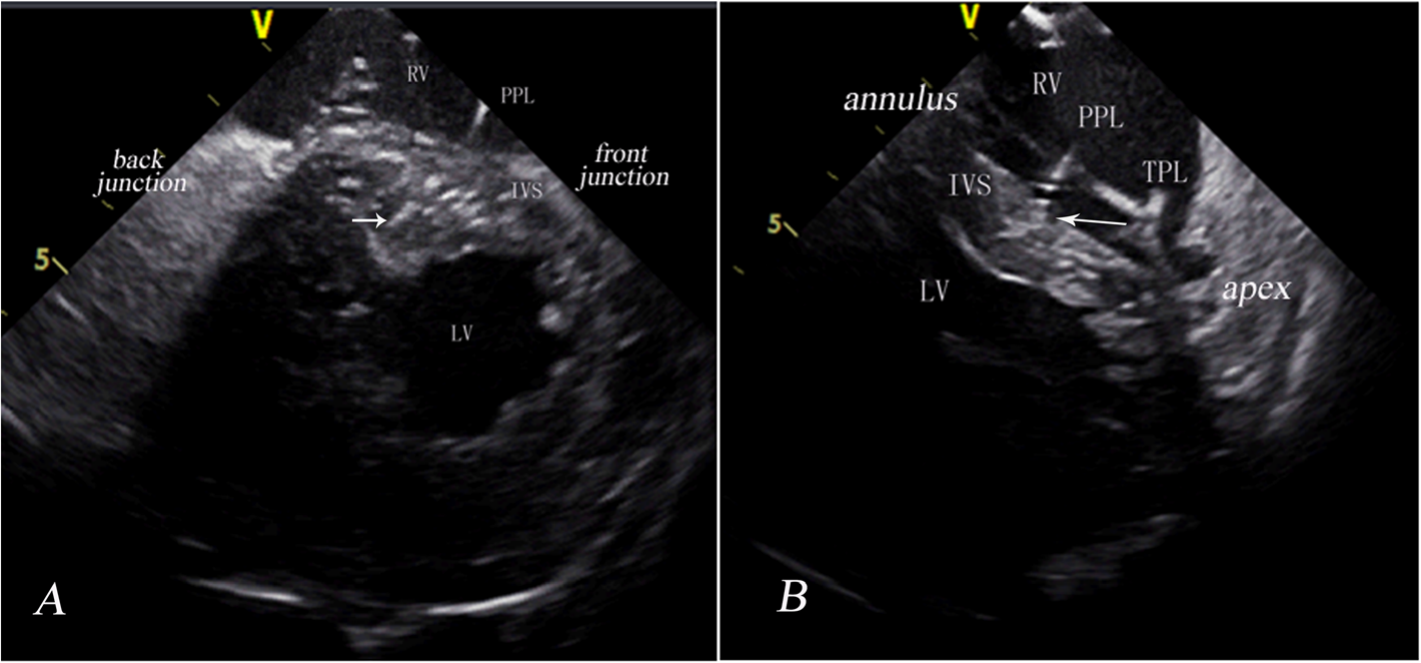
Intracardial echocardiography views obtained with the catheter in the RV. **a**: Short axis view of the septum around the mitral valve plane. The distance between the implantation site and the front junction point and the distance between the implantation site and the back junction point should be measured. **b**: Longitudinal view of the septum. The distance from the implantation site to the apex and the distance from the implantation site to the tricuspid annulus should be measured. RV, right ventricle; LV, left ventricle; V, transducer location; IVS, interventricular septum; 5, distance to the transducer; PPL, permanent pacing lead; TPL, temporary pacing lead; short arrow, lead tip; long arrow, implantation site

When the catheter passes through the TV, the “P” flex is released gently to position the tip close to RVOT. Then, the catheter is gradually rotated counterclockwise to image a short axis view of the LV and IVS. The LV, RV and IVS should be visualized in both the short axis around the mitral plane and a longitudinal view of the LV (Fig. 1).

### Anatomical localization

#### The summit site of the tricuspid annulus

**Fig. 2:**
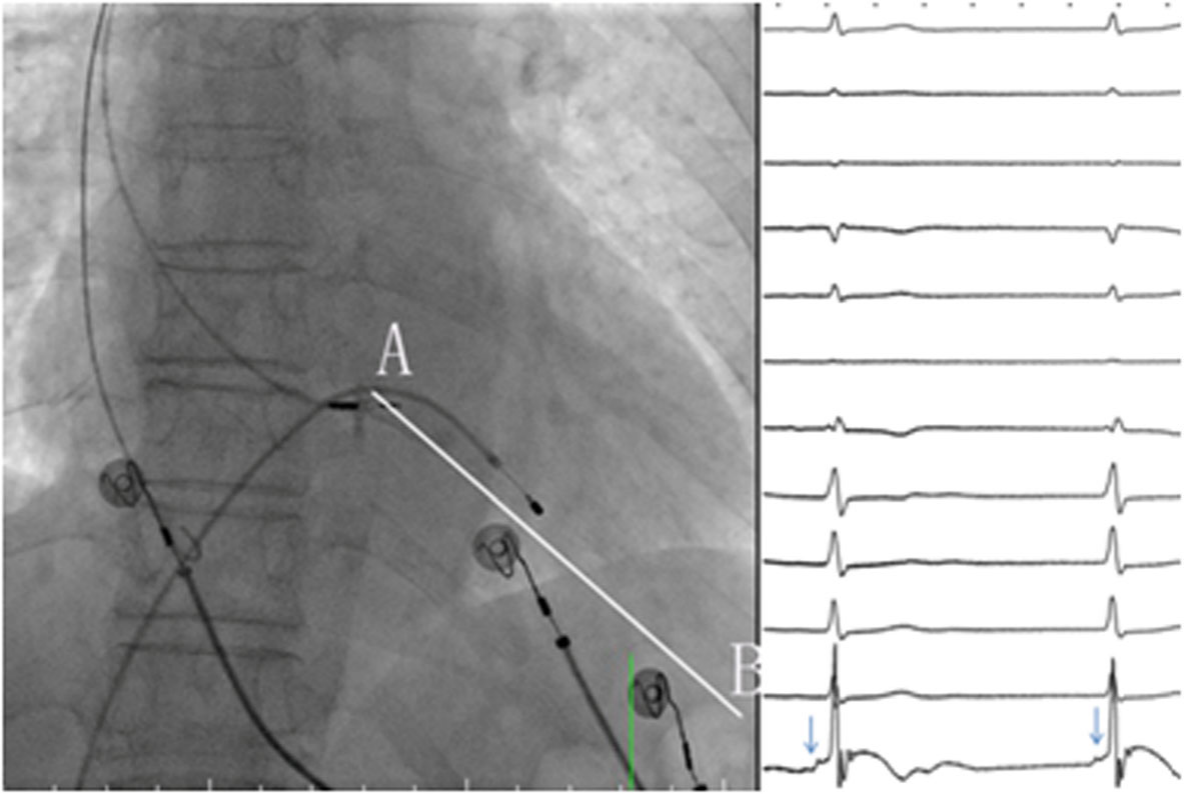
Fluoroscopic image of RV and His bundle potential. Left plane: fluoroscopic image of RV at the RAO 30° projection. **a**, the summit site of the tricuspid annulus; **b**, RV apex. Right plane: inter-cardiac electrocardiogram of the lead. When the lead tip was at the summit site, the His bundle potential (arrows) was shown. RV, right ventricle

**Fig. 3:**
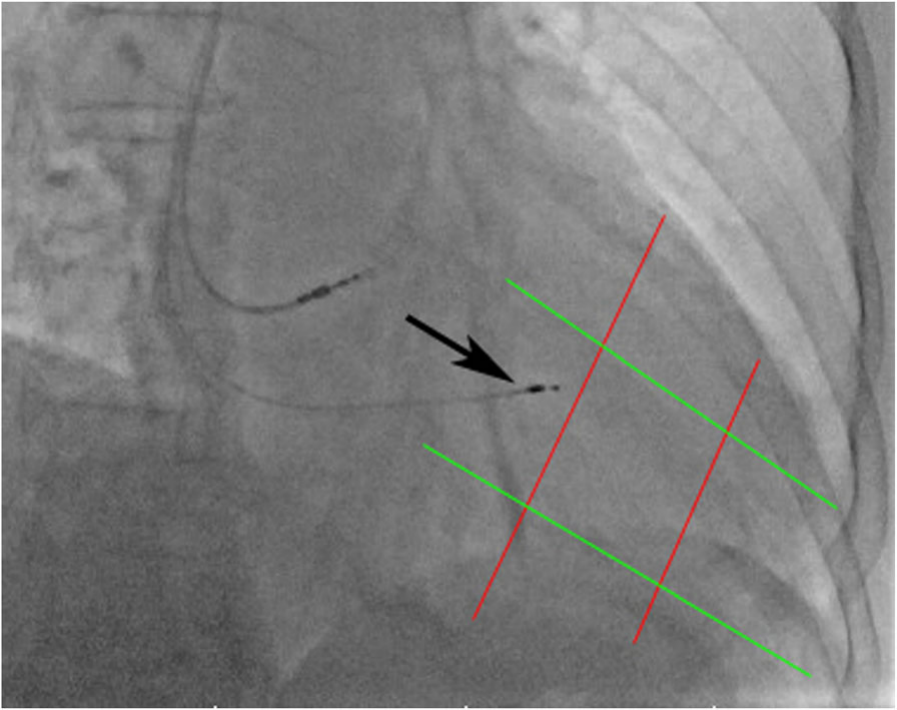
Fluoroscopic image of the LBBAP area in the RAO 30° projection. Two parallel lines along the long axis plane (green lines) and the short axis plane (red lines) divided every plane into three parts. The RV in this plane was divided into nine parts. The arrow indicates the mid-basal region. LBBAP, left bundle branch area pacing; RV, right ventricle

There is no true bifurcation of the bundle of His in the human heart [12]. The summit site of the tricuspid annulus is close to the His bundle (Fig. 2, right plane) and can be used as an anatomical marker. The C315 sheath is first advanced into RVOT over a long guide wire. When the guide wire is withdrawn, the tip of the sheath is in direct contact with the septal tissue. The 3830 pacing lead is then advanced towards the tip of the sheath. The sheath is pulled back towards RA by minimal clockwise rotation. From the jump sign, we found the summit site of the tricuspid annulus and marked it in the fluoroscopy image in the right anterior oblique (RAO) 30° projection (Fig. 2, left plane). Then, the C315 sheath is moved forward 1.0–2.0 cm from the summit site towards the apex in the RAO 30° projection (Fig. 2, left plane) or in the mid-basal region of the nine-grid system (Fig. 3), where a paced QRS morphology of the ‘W’ pattern is noted.

#### Nine-grid system

Single-plane right ventricle fluoroscopy in RAO 30° projection was performed, and the images of fluoroscopy were reviewed. The RV end-diastolic images were determined. Then, two parallel lines along the long axis plane and the short axis plane were drawn, which divide every plane into three parts, and the distance between two adjacent lines was equal. The RV in this projection was divided into nine grids, and the desired implantation site was proposed as the intersection region between the posterior part of the long axis plane and the middle part of the short axis (mid-basal region) [18] (Fig. 3).

### Pacing localization

**Fig. 4:**
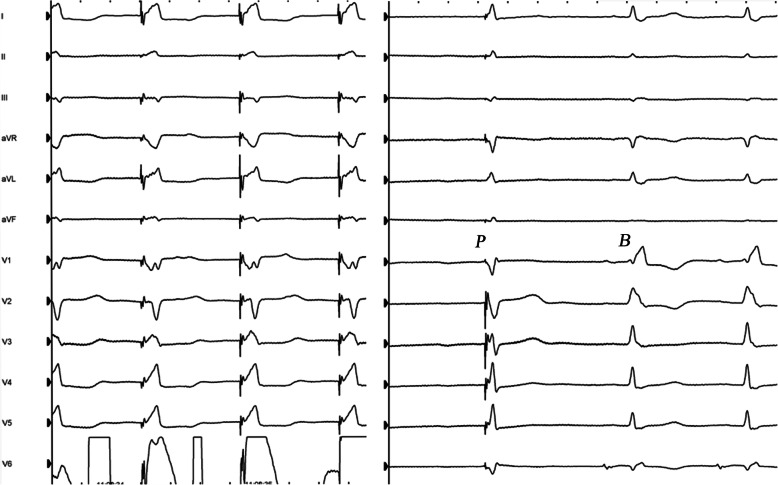
The paced ECG at the ideal implantation site. Left plane: the paced ECG at the ideal implantation site. During unipolar tip pacing, the paced QRS complex in lead V_1_ displayed a “W” morphology. Bidirectional waves were visible in one of the inferior wall leads. Right plane: baseline ECG (B) and bipolar paced ECG (P) at LBB. The baseline ECG showed a complete RBBB morphology with a QRSd of 156 ms that was corrected by bipolar LBBAP to a narrow QRSd of 116 ms. LBBAP, left bundle branch area pacing

Pace mapping at 3 V and 0.4 ms was performed to identify the ideal implantation site during unipolar tip pacing (UTP) with the following criteria. The paced QRS complex in lead V_1_ should display a “W” morphology with a mid-notch close to the bottom [16]. Bidirectional waves should be visible in one of the inferior wall leads (Fig. 4). The paced QRS duration at 10 V/0.4 ms should be less than at 3 V/0.4 ms.

### Two-way monitoring

When screwing the lead, it is important to keep the sheath perpendicular to the ventricular septum and remain stable in one direction such that the lead is vertical directly against the septum in the LAO 45° view and points towards the 12–1 o’clock angle in the RAO 30° view (two-way monitoring) [19].

The unipolar pacing impedance should be gradually decreased but not less than 500 Ω. If it is less than 500 Ω or dropping too fast, one should consider the risk of lead perforation. The significant rise in unipolar pacing impedance above 900 Ω generally suggests that the lead is penetrating in an oblique direction and may need to be reoriented [19].

As the depth increased, the notch in the paced QRS complex in lead V_1_ gradually migrated towards the end of the QRS wave, and the QRS duration narrowed together with the appearance of the paced QRS morphology with the RBB block (RBBB) pattern in lead V_1_ and the S wave in lead V_5_ and V_6_ [20].

Left ventricular activation time (LVAT) in unipolar lateral precordial leads V_4_-V_6_ is defined as the stimulus to the peak of the R wave. With increasing depth, LVAT should become shorter, suggestive of LBB capture [21].

### LBBAP success criteria

According to a previous study [16, 22], successful LBBAP should be characterized as capturing the LBB with or without myocardial capture, with a narrow RBBB morphology. A discrete component between the pacing stimulus and ventricular activation in the intracardiac electrograms could be recorded at different pacing outputs. The left bundle branch potential could be recorded at or near the LBB area. The pacing impedance should be > 500 Ω, the QRS complex duration should be ≤130 ms [23], the pacing threshold should be less than 1.5 v, and the LBB injury current should be high during UTP. Once the above criteria were met, the lead tip was located at or near the LBB, and the lead advancement was stopped. Then, ICE was used to assess the results in sequence. If successful LBBAP could not be achieved after five attempts of lead positioning, the lead should then be placed in the mid-LV septum by transseptal access to achieve a relatively narrow QRS duration, namely, LV septum pacing [14].

### ICE confirmation

At the short axis view around the mitral valve (Fig. 1A), the distance between the implantation site and the front junction point of the left-right ventricle (the upper) and the distance between the implantation site and the back junction point of the left-right ventricle (the lower) were measured during diastole. Then, the ratio of the upper to the lower was calculated. Less than 1 is considered the upper part of the septum, and greater than 1 is considered the lower part of the septum.

In the longitudinal view (Fig. 1B), the distance from the implantation site to the apex (the front) and the distance from the implantation site to the tricuspid annulus (the back) were measured during diastole. The ratio of the front to the back was calculated. Greater than 1 refers to the posterior part of the septum, while less than 1 refers to the anterior part of the septum. The posteromedial basal septum is proposed as the ideal implantation target site, which means that the ratio should be > 1 at the short axis plane around the tricuspid annulus and ≈2 at the longitudinal view. The distance between the tip of the lead and endocardium of LV-IVS was also measured.

#### Clinical follow-up

Patients were seen for routine clinical follow-up at standard time periods (1 month, 3 months, 6 months, and 12 months). Functional status was assessed using NYHA classification. Device thresholds were checked and adjusted as needed to maximize battery longevity. The pacing threshold, impedance and R wave sensing were measured. According to previous literature [24], a high pacing threshold at baseline was defined as a pacing threshold over 2.5 V/0.4 ms, increased threshold over 1.0 V compared with the baseline after the procedure and at follow-up. Echocardiography was performed in cases clinically indicated during follow-up.

### Statistical analysis

Continuous variables were given as the mean ± SD or median. Paired comparisons were made using Student’s t test if the data were normally distributed and using the Wilcoxon signed-rank test for nonparametric data. Paired categorical data (NYHA functional class) were compared using the Wilcoxon test. *P* ≤ 0.05 was considered statistically significant.

## Results

A total of 15 patients [mean age: 69.6 ± 11.8 years (28–85), 7 male] referred for primary pacemaker implantation and underwent LBBAP procedures with our proposed method during the study period were included. Among 15 patients, LBBAP was successfully achieved and demonstrated an RBBB pattern during UTP in 13 patients. The acute success rate was 86.7% (13/15), which demonstrated that our method is a useful method for assisting with lead implantation. Of two unsuccessful patients, the RBBB pattern disappeared during C315 sheath removal in 1 patient, and another patient with baseline RBBB received LV septum pacing with a paced QRSd > 130 ms after failed LBBAP 5 times. All procedures were performed under the guidance of ICE. The operation duration was 133 ± 32.9 min. The time of X-ray fluoroscopy was 21.2 ± 5.9 min. The mean time for 3830 lead positioning during LBBAP was 33.8 ± 16.6 min.

### Baseline characteristics

Among 15 patients, three remained in sinus bradycardia. Sinus pause-dependent atrial fibrillation was noted in 1 patient. Atrioventricular block was present in the remaining 11 patients (second degree in 4 patients; high degree in 3; third degree in 4). The left ventricular ejection fraction (LVEF) was 62.6 ± 10.5%, and a reduction in LVEF was found in two patients (25 and 34%). Three patients (20.0%, 3/15) had histories of myocardial infarction or percutaneous coronary intervention, 66.7% (10/15) had hypertension, and 20.0% (3/15) had diabetes mellitus. Baseline left bundle branch block (LBBB) (*n* = 3) or RBBB (*n* = 3) was present in 6 patients (40.0%, 6/15).

ECG characteristics and pacing parameters.

Among 15 patients, the baseline QRS duration was 110.4 ± 33.1 ms. After unipolar LBBAP, 13 patients demonstrated an RBBB pattern with a paced QRSd of 114.1 ± 16.1 ms (*P* > 0.05 vs. baseline). Among 13 LBBAP patients, LBB potential could be recorded in 10 patients from the LBB lead (10/13, 76.9%), and the mean interval of LBB potential to the beginning of the QRS complex was 29.5 ± 4.4 ms. The LVAT for all LBBAP patients was 76.2 ± 8.6 ms, and the R wave sensing amplitude, pacing impedance, and unipolar pacing capture threshold were 9.5 ± 2.7 V, 845 ± 106.4 Ω, and 1.08 ± 0.86 V/0.4 ms respectively after implantation (Table 1).

**Table 1:** Pacing characteristics in patients with successful LBBAP during UTP (mean ± SD)

	Pacing threshold(v/0.4 ms)	Pacing impedance (Ω)	R wave sensing (mV)
In procedure	1.08 ± 0.86	845 ± 106.4	9.5 ± 2.7
1 month after procedure	0.92 ± 0.18	761 ± 62.3	12.4 ± 2.2
3 months after procedure	0.82 ± 0.22	916 ± 123.4	13.4 ± 4.6
*P* value	0.316	0.458	0.576

*LBBAP* Left bundle branch area pacing, *UTP*, Unipolar tip pacing

For patients with complete LBBB (*n* = 3) or RBBB (*n* = 3), LBBAP corrected both the LBBB (*n* = 3) and the RBBB (*n* = 2) with a successful bundle branch correction rate of 83.3%. The RBBB morphology could be corrected by bipolar LBBAP at a low output. In the case shown in Fig. 4 (right plane), the baseline ECG showed a complete RBBB morphology with a QRSd of 156 ms, which was corrected by bipolar LBBAP to a narrowed QRSd of 116 ms.

### ICE measurements

The posteromedial basal septum was clearly visualized by ICE. The front distance and the back distance were 33.4 ± 10.6 and 24.5 ± 10.2 mm, respectively, and the ratio of the front to the back was 1.76 ± 1.36. The upper distance and the lower distance were 44.9 ± 10.7 and 33.2 ± 10.4 mm, respectively. The ratio of the upper to the lower was 1.57 ± 0.80 (Table 2). These results suggested that the implantation site for LBBAP was at the posteromedial basal septum.

**Table 2:** ICE parameters of lead tips in patients with successful LBBAP (mean ± SD)

	short axis around the mitral plane	longitudinal views
upper / front distance (mm)	33.4 ± 10.6	44.9 ± 10.7
Lower / back distance (mm)	24.5 ± 10.2	33.2 ± 10.4
Ratio(−--)	1.76 ± 1.36	1.57 ± 0.80
The distance between tip and left IVS endocardium (mm)	2.22 ± 1.75	–

*ICE* Intracardiac echocardiogram, *LBBAP* Left bundle branch area pacing

**Fig. 5:**
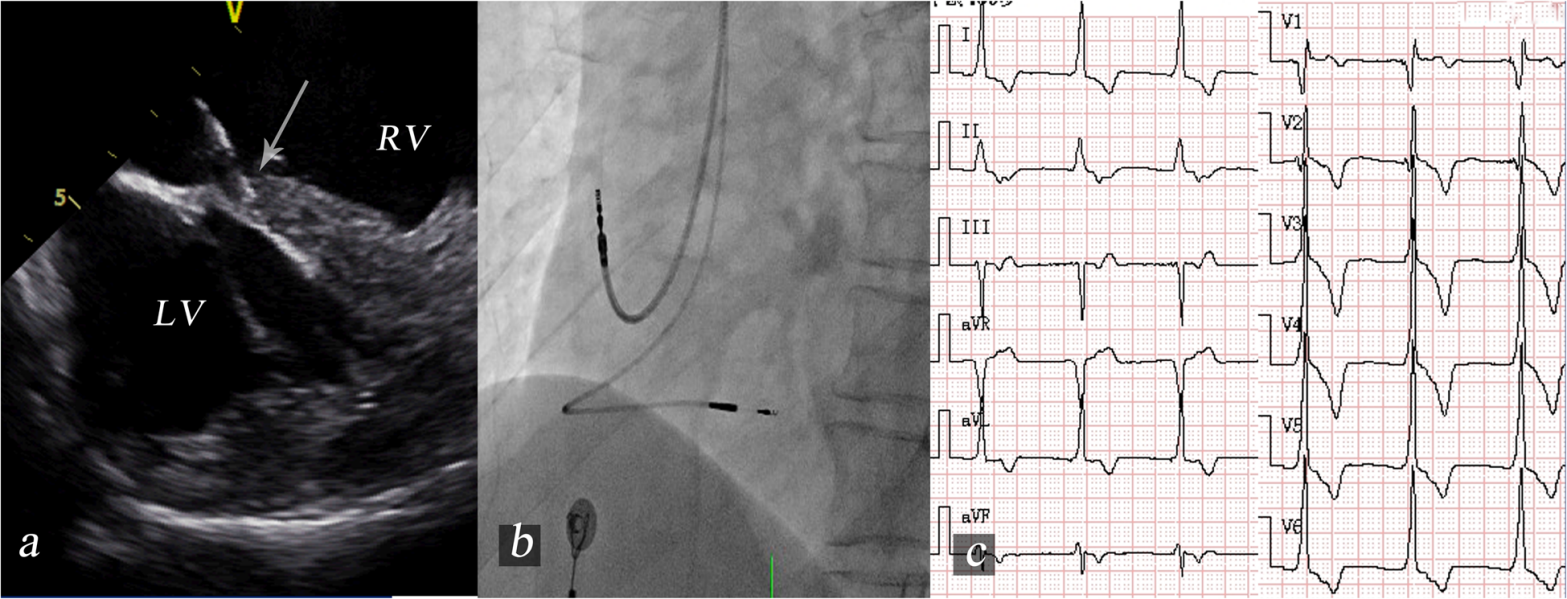
The images of a failed lead implant with dislodgement after 3 months. **a**: Intracardiac echocardiography image. The 3830 lead was delivered into the IVS but was nonperpendicular (arrow). **b**: Fluoroscopic left anterior oblique projection. This plane showed the final lead position in the IVS. **c**: The unipolar tip paced ECG. During unipolar tip pacing, “RBBB” morphology with 120 ms of QRS duration was displayed in lead V_1_ but disappeared after C315 sheath removal. RV, right ventricle; LV, left ventricle; V, transducer location; IVS, interventricular septum; 5, distance to the transducer; RBBB, right bundle branch block

Among 13 LBBAP patients, the distance between the lead tip and left side of the IVS was 2.22 ± 1.75 mm. Of the two patients with failed LBBAP, the above distances were 3.4 mm and 1.9 mm, respectively. ICE results showed that when screwing the pacing lead, the tip deviated from the original direction in an oblique direction (Fig. 5).

### Follow-up

Overall, all 13 successful LBBAP patients maintained the same pacing characteristics during the 1-month follow-up period, and the latest success rate was 13/15 (86.7%). During the 3-month follow-up period, pacing parameters, including the sensing amplitude, pacing threshold and impedance, remained stable in 12 patients (all, *P* > 0.05) (Table 1), except that one patient had an increase in the LBB capture threshold to 3.0 v/0.4 ms. The paced QRSd still remained narrow at a 2.0 V at 0.4 ms output. Of two failed patients, lead dislodgement developed during follow-up at 3 months in one patient (Fig. 5), and lead revision was performed; the other remained stable.

Transthoracic echocardiogram evaluation data at baseline and 3-month follow-up were available in 13 patients receiving successful LBBAP (Table 3). As shown in Table 3, in patients with successful LBBAP, the left ventricular end-diastolic dimension (50.9 ± 6.3 mm vs. 50.0 ± 8.0 mm, *P* > 0.05) and LVEF (62.6 ± 10.5% vs. 61.4 ± 10.4%, *P* > 0.05) were similar between the 3-month follow-up and baseline.

**Table 3:** Comparison of the in- and postoperation TTE parameters (mean ± SD)

	LVEDD (mm)	LVEF (%)	QRS duration (ms)
Before procedure	50.9 ± 6.3	62.6 ± 10.5	110.4 ± 33.1
3 months after procedure (UTP)	50.0 ± 8.0	61.4 ± 10.4	114.1 ± 16.1
*P* value	0.580	0.651	0.467

*TTE* Transthoracic echocardiogram, *UTP* Unipolar tip pacing, *LVEF *Left ventricular ejection fraction, *LVEDD* Left ventricular end-diastolic dimension

#### Safety assessment

Coronary angiography was performed in the first 5 LBBAP patients, and no coronary injury was observed during LBBAP implantation. Only one patient had an increase in the LBB capture threshold to 3.0 v/0.4 ms at 3 months without dislodgement, as confirmed by X-ray, and no patient had an increase in the threshold of > 1.0 v. Apart from one of two failed patients, other patients showed no signs of dislodgement, loss of capture, infections, embolism, or stroke associated with the implantation.

## Discussion

LBBAP is a physiological pacing method and can be achieved by screwing the 3830 lead deep enough into the IVS and capturing the left bundle branch conduction system. However, the RV implantation site remains elusive. The present study demonstrated that a combination of a nine-grid system on fluoroscopy and the use of ICE is helpful for guiding lead implantation to the desired site. The present study also showed that LBBAP can be achieved by screwing the 3830 lead deep into the posteromedial basal septum and pacing both the LBB and adjacent ventricular tissues.

Using our methods, assisted by pacing localization, we could quickly locate the desired implantation site at the RV septum and avoid damaging the His bundle system. The two-way monitoring method, with the help of the notch in the paced QRS complex in lead V_1_, LVAT in lead V_5_, could monitor and control the lead orientation in the septum effectively. The mean time for 3830 lead positioning during LBBAP was 33.8 ± 16.6 min, which was similar to previous reports (31.4 ± 14.1 min) [8]. This demonstrated that our method could be a useful method for assisting with lead implantation. The duration of operation time and the time of X-ray fluoroscopy were 133 ± 32.9 and 21.2 ± 5.9 min, respectively, both of which were longer than those in a previous report (117 ± 48 and 16.4 ± 12.3 min) [25], which suggested that unskillful handling of ICE could extend the procedure time and fluoroscopy time. The acute success rate was 86.7%. This verified that our methods used in this study were clinically feasible.

To date, the precise and ideal anatomical location of LBBAP, which should theoretically be located distal to the His bundle, trunk of the LBB, or proximal to the left anterior/posterior fascicle bundle, has not yet been well elucidated. The LBB potential, a determinant of LBB capture, confirms the pacing site at the left bundle branch or nearby the left bundle branch. The threshold differences between LBB capture and myocardial capture may be too close to discriminate [21]. To avoid perforation, recording LBB potential may not be necessary [15], as long as the bundle branch is stimulated, leading to fast conduction with normal or near-normal ECG. Narrow QRS duration (≤130 ms), RBBB pattern, and left posterior or anterior fascicular block morphology are features of successful LBBAP [21]. The present study showed that the paced QRS duration was 114.1 ± 16.1 ms and the mean LVAT was 76.2 ± 8.6 ms, and the LBB potential was found in 76.9% of patients, which suggested that LBBAP was successful.

The use of ICE allowed us to monitor all procedures. The procedure was successfully completed without acute complications, and LBBAP electrocardiogram post procedure was presented in the 13 patients. ICE showed that the ratio of the front to the back was 1.57 ± 0.80, and the ratio of the upper to the lower was 1.76 ± 1.36, suggesting that the implantation site was at the posteromedial basal septum. ICE was also helpful in defining the aetiology of failed LBBAP procedures. In our two failed patients, the lead all deviated from the original direction, even though the tip moved back in a patient.

Our experience suggests that ICE guidance may have the following merits. ① The direct visualization of endocavitary structures, which is currently not provided by any other real-time mapping system. ② Monitoring of the lead orientation in the septum during fixing. ③ Direct visualization of the depth of the tip into the septum reduces the risk of perforation. However, unskillful handling of ICE could also interfere with the lead, even pulling it back.

Considering both pacing efficiency and safety, a series of tips and tricks for lead implantation may be shared as follows. Heparinized saline flush pre-procedures are used to prevent thrombus formation within the C315 sheath lumen. A guide wire (0.035 in*120 cm, J-tip) is available and easily guides the C315 tip to the desired site. A suitable power is needed to keep the C315 tip perpendicular to the IVS. A combination of using the two-way monitoring method and ICE could identify the lead orientation in the septum and avoid deviating from the original direction. The 3830 lead depth should be carefully estimated during implantation to avoid passing through IVS.

Some limitations need to be discussed. First, ICE is expensive, which is linked with increased medical cost. Second, the present study had a short follow-up interval. We expect long-term favourable clinical benefits, as observed in the case reported by Huang et al. [26]. Third, it is unknown whether the clinical efficacy of LBBAP with an appropriate AV delay would be the same as or better than LV epicardial pacing or cardiac resynchronization therapy. Fourth, large femoral venous access would also be an added concern. Fifth, there was no control group in this study.

## Conclusions

Our preliminary results indicate that the posteromedial basal septum could be seen as the implantation target site for LBBAP. As a technique for LBBAP, ICE is a useful method for assisting with lead implantation. It is feasible and safe to use a nine-grid system combined with ICE for LBBAP.

## Data Availability

Data are available from the corresponding author upon reasonable request due to privacy or other restrictions.

## Abbreviations

LBB/AP: Left bundle branch/area pacing
IVS: Interventricular septum
AV: Atrioventricle
RV/OT: Right ventricle/outlet tract
ICE: Intracardiac echocardiogram
TV: Tricuspid valve
RA: Right atrium
LV: Left ventricle
RAO: Right anterior oblique
LAO: Left anterior oblique
UTP: Unipolar tip pacing
LVAT: Left ventricular activation time
LVEF: Left ventricular ejection fraction
LBBB: Left bundle branch block
RBBB: Right bundle branch block
QRSd: QRS duration
